# J-curve relationship between long term glycemic control and mortality in diabetic patients with acute myocardial infarction undergoing percutaneous coronary intervention

**DOI:** 10.1186/s12933-021-01428-x

**Published:** 2021-12-15

**Authors:** Ik Jun Choi, Eun Ho Choo, Hwa Jung Kim, Sungmin Lim, Donggyu Moon, Kwan Yong Lee, Byung-Hee Hwang, Chan Joon Kim, Mahn-Won Park, Jong-Min Lee, Chul Soo Park, Hee-Yeol Kim, Ki-Dong Yoo, Doo Soo Jeon, Wook Sung Chung, Min Chul Kim, Myung Ho Jeong, Youngkeun Ahn, Kiyuk Chang

**Affiliations:** 1 Division of Cardiology, Department of Internal Medicine, Incheon St. Mary’s Hospital, College of Medicine, The Catholic University of Korea, Seoul, Republic of Korea; 2 Catholic Research Institute for Intractable Cardiovascular Disease (CRID), College of Medicine, The Catholic University of Korea, Seoul, Republic of Korea; 3 Division of Cardiology, Department of Internal Medicine, Seoul St. Mary’s Hospital, College of Medicine, The Catholic University of Korea, 222, Banpo-daero, Seocho-gu, Seoul, 06591 Republic of Korea; 4 Division of Cardiology, Department of Internal Medicine, Uijeongbu St. Mary’s Hospital, College of Medicine, The Catholic University of Korea, Seoul, Republic of Korea; 5 Division of Cardiology, Department of Internal Medicine, St Vincent’s Hospital, College of Medicine, The Catholic University of Korea, Suwon, Republic of Korea; 6 Division of Cardiology, Department of Internal Medicine, Daejeon St. Mary’s Hospital, College of Medicine, The Catholic University of Korea, Daejeon, Republic of Korea; 7 Division of Cardiology, Department of Internal Medicine, Yeouido St. Mary’s Hospital, College of Medicine, The Catholic University of Korea, Seoul, Republic of Korea; 8 Division of Cardiology, Department of Internal Medicine, Bucheon St. Mary’s Hospital, College of Medicine, The Catholic University of Korea, Seoul, Republic of Korea; 9 Division of Cardiology, Department of Internal Medicine, Chonnam National University Hospital, Chonnam National University School of Medicine, Gwangju, Republic of Korea

**Keywords:** Diabetes mellitus, Glycated hemoglobin A, Myocardial infarction, Mortality, Hypoglycemia

## Abstract

**Background:**

Intensive glycemic control is generally recommended for diabetic patients to reduce complications. However, the role of glycemic control in the mortality in diabetic patients with acute myocardial infarction (AMI) remained unclear.

**Methods:**

We selected diabetic patients who measured HbA1c more than 3 times after AMI among 10,719 patients enrolled in the multicenter AMI registry. Patients (n = 1384) were categorized into five groups: according to mean HbA1c level: ≤ 6.5%, > 6.5 to ≤ 7.0%, > 7.0 to ≤ 7.5%, > 7.5 to ≤ 8.0% and > 8.0%. The primary endpoint was all-cause mortality.

**Results:**

During a median follow-up of 6.2 years, the patients with a mean HbA1c of 6.5 to 7.0% had the lowest all-cause mortality. Compared to patients with mean HbA1c of 6.5 to 7.0%, the risk of all-cause mortality increased in subjects with mean HbA1c ≤ 6.5% (adjusted hazard ratio [HR] 2.00, 95% confidence interval [CI] 1.02–3.95) and in those with mean HbA1c > 8.0% (adjusted HR 3.35, 95% CI 1.78–6.29). In the subgroup analysis by age, the J-curve relationship between mean HbA1c and all-cause mortality was accentuated in elderly patients (age ≥ 65 years), while there was no difference in all-cause mortality across the HbA1c groups in younger patients (age < 65 years).

**Conclusions:**

The less strict glycemic control in diabetic patients with AMI would be optimal for preventing mortality, especially in elderly patients.

**Supplementary Information:**

The online version contains supplementary material available at 10.1186/s12933-021-01428-x.

## Background

Therapeutic strategies to manage blood pressure, lipid, and glucose levels have evolved in the past two decades to reduce the increased risk of macrovascular and microvascular complications in diabetic patients. Although lowering blood pressure and lipid levels were demonstrated the benefit for the decrease of macrovascular complications or cardiovascular (CV) events, the relationship between intensive glucose control and CV outcomes is still uncertain. Several randomized controlled trials have investigated the effect of intensive glucose control on CV disease, which showed positive effects on some CV outcomes, but controversial results for the risk of CV death and all-cause death [1–5]. However, meta-analyses suggested that an increase of 1% in the glycated hemoglobin level (HbA1C), reflecting blood glucose levels over the preceding 2 to 3 months, was associated with an 18% increased risk of CV events and a 15% increase in the risk of death [6–8].

The current guidelines recommend that intensive glucose control targeting HbA1c ≤ 6.5% to 7.0%, but HbA1c targets shall be individualized according to patients’ conditions [9–11]. Because intensive glucose control inevitably accompanies an increased risk of experiencing hypoglycemic events associated with increased mortality [12, 13]. One of the mechanisms of hypoglycemia-induced mortality would be the surge of adrenergic hormones, which may result in the increased risk of vasoconstriction, thrombogenesis, and ventricular arrhythmia [14]. Hypoglycemic events may be more dangerous for patients already diagnosed with CV disease. Considering that patients with acute myocardial infarction (AMI) should be treated with beta-adrenergic blockers to reduce subsequent CV events, the hypoglycemic event can increasingly affect diabetic patients with AMI. Though the current guidelines recommend less-stringent HbA1C goals for patients with multiple comorbidities and elderly patients, evidence regarding the HbA1c target for AMI patients with diabetes is limited. Thus, the present study evaluated the effect of intensive glucose control on mortality in diabetic patients with AMI. We also investigated whether the result of intensive glucose control differs according to patients’ age.

## Methods

### Study population

The COREA-AMI (CardiOvascular Risk and idEntificAtion of potential high-risk population in Acute Myocardial Infarction) registry was designed to evaluate real-world, long-term clinical outcomes in all patients with AMI. AMI was diagnosed by detecting elevated cardiac biomarkers at least 1 value above the 99th percentile upper reference limit with a temporal rise and fall and at least 1 of the following indications: symptoms of ischemia, new or presumed new significant ST-segment-T wave changes, or new left bundle branch block, development of pathological Q waves on electrocardiography, imaging evidence of new viable myocardium loss or a new regional wall motion abnormality, or an intracoronary thrombus identified by angiography. According to electrocardiography findings, the clinical presentation was divided into ST-segment elevation myocardial infarction (STEMI) and non-ST-segment elevation myocardial infarction (NSTEMI). The COREA-AMI I registry has been previously reported and included patients with AMI who underwent percutaneous coronary intervention (PCI) from January 2004 to December 2009. The COREA-AMI II registry included additional patients from January 2010 to August 2014. The current registry comprised updated new clinical and angiographic parameters and evaluated long-term clinical follow-up data for as long as possible until 2019. The study was conducted in compliance with the Declaration of Helsinki regarding investigations in humans. The study protocol was approved by the Institutional Review Board at participating centers. This registry has been registered on ClinicalTrials.gov (study ID: NCT02806102).

**Fig. 1:**
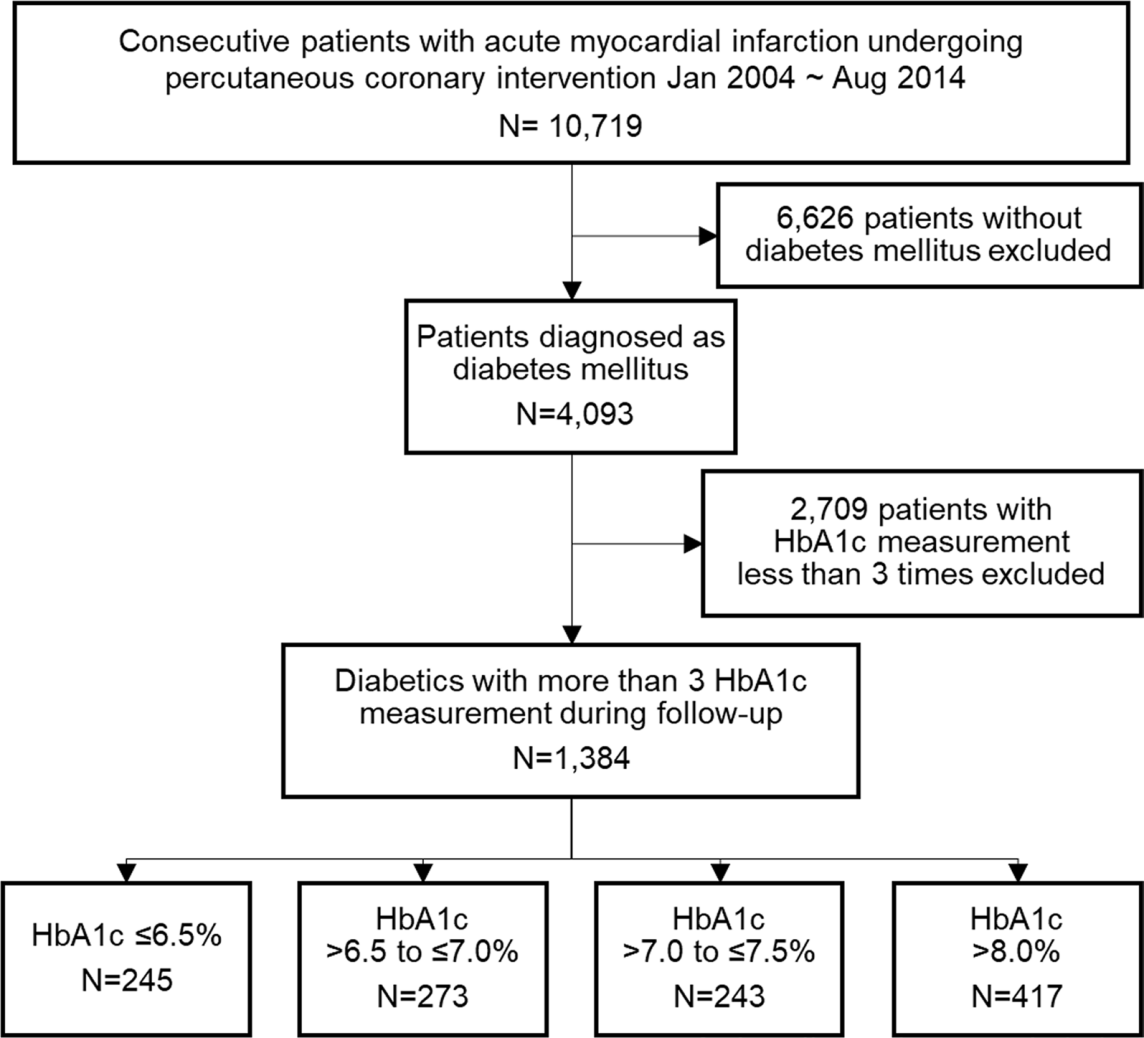
Study population. Flow chart outlining the selection of the study population

Figure 1 outlines the study flow. Of the 10,719 patients in the COREA-AMI registry, 4093 had diabetes mellitus at index hospitalization. Diabetes mellitus was diagnosed if they had diabetes diagnosed by a physician, if they were taking insulin or oral hypoglycemic agents, or if HbA1c ≥ 6.5% or random glucose ≥ 200 mg/dL was observed at index hospitalization. We selected 1384 patients who had undergone at least three measurements of HbA1c during follow-up. The patients were categorized into five groups according to mean HbA1c level during follow-up period in 0.5% interval: ≤ 6.5%, > 6.5 to ≤ 7.0%, > 7.0 to ≤ 7.5%, > 7.5 to ≤ 8.0% and > 8.0%.

### Study outcomes

The primary outcome of this analysis was all-cause mortality. The secondary endpoints were cardiovascular death, MI, and stroke. All-cause deaths were attributed to cardiovascular events unless a non-cardiac death could be clearly identified. Recurrent MI was defined as the presence of recurrent symptoms and new electrocardiographic changes compatible with MI or cardiac markers expressed at least two-fold above the normal limit. Stroke was defined as the presence of a new focal neurologic deficit thought to be vascular in origin, with signs or symptoms lasting more than 24 h.

### Data collection

All data were collected on a web-based system after eliminating personal information. Patients’ follow-up data, including survival data and clinical event data, were collected through March 31, 2019, via hospital chart reviews and telephone interviews of patients conducted by trained reviewers who were blinded to the study results. Independent reviewers and interventional cardiologists assessed angiographic and procedural data, and independent research personnel collected baseline clinical, laboratory, and medication data. Clinical events and outcome data were assessed by electrical medical records and telephone conversations. All adverse clinical events of interest were confirmed centrally by the committee of the Cardiovascular Center of Seoul St. Mary’s Hospital, Seoul, Republic of Korea. Validation for mortality was performed on the basis of disqualification from the National Health Insurance Service, which is the single government-managed insurance and covers almost all of the nation’s population. The final dataset was handled by independent statisticians of the Clinical Research Coordinating Center and sealed with a code by the Clinical Research Associate.

### Statistical analysis

Baseline characteristics were expressed as the mean (SD) and analyzed statistically using ANOVA. Categorical variables were expressed as numbers (and percentages or rates) and analyzed using the Chi‐square test or Fisher’s exact test, as appropriate. Kaplan–Meier survival curves were constructed to examine the cumulative incidence of primary and secondary outcomes; the incidence rates of outcomes were compared using the log-rank test. A Cox proportional-hazards regression model was used to calculate estimates of relative risk and 95% confidence intervals (CI) between each of the five groups. In addition to crude hazard ratios (HRs), adjusted HRs were estimated after adjustment for potential confounding factors. The model was adjusted using three models. Model 1 was adjusted for age, sex, and body mass index. Model 2 was adjusted for model 1 plus systolic blood pressure, hypertension, current smoke, chronic kidney disease, and previous stroke. Model 3 was adjusted for model 2 plus the type of MI (STEMI or NSTEMI), left ventricular ejection fraction, low-density lipoprotein cholesterol, Global Registry of Acute Coronary Events (GRACE) score, and complete revascularization. The unadjusted and adjusted hazard ratios for each category of HbA1c were calculated in reference to the HbA1c of 6.5% to 7.0%, where the hazard ratio was considered as 1. In addition, nonlinear Cox proportional hazards models were estimated with mean HbA1c as a continuous variable and with the square of HbA1c. We performed subgroup analysis by age to assess whether the target HbA1C goal would be less stringent for elderly patients. We divided the study population by the age of 65 years and compared the relationship between HbA1c and mortality. Statistical analyses were conducted with R version 4.0.2 (R Foundation for Statistical Computing, Vienna, Austria). All the statistical testing was 2-sided. p-value < 0.05 was considered statistically significant.

## Results

### Baseline characteristics

The mean age of the 1384 patients was 60 ± 11 years, and 1012 (73.1%) were men. During the median follow-up period of 6.2 years (interquartile range: 4.6–7.9), the median number of HbA1c measurements was 4 (interquartile range: 3–6). The median interval between HbA1c measurements was 470 days. The mean HbA1c was 7.6 ± 1.2 % overall. Baseline characteristics of the entire cohort and the patients categorized by HbA1c classification are depicted in Table 1. Patients with lower HbA1c were older and more likely to have hypertension, a history of stroke, higher left ventricular ejection fraction, and lower cholesterol levels. Though patients with lower HbA1c had a lower estimated glomerular filtration rate, the proportion of severe and moderate chronic kidney disease was not different among the groups. Patients with lower HbA1c were less likely to be treated with insulin, metformin, and sulfonylurea. The Geriatric Nutritional Risk Index (GNRI) of patients with ≤ 6.5%, patients with 7–7.5%, and patients with HbA1c > 8.0% were similar, and only GNRI of patients with HbA1c > 8.0% was significantly lower only in compared to that of the patients with HbA1c of 6.5 to 7.0%. Angiographic findings, including the severity, extent of coronary artery disease, and the degree of stent implantation, were similar among the groups.

**Table 1:** Baseline characteristics of the patients according to the glycated hemoglobin level

	HbA1c ≤ 6.5% (N = 245)	6.5% < HbA1c ≤ 7.0% (N = 273)	7.0% < HbA1c ≤ 7.5% (N = 243)	7.5% < HbA1c ≤ 8.0% (N = 206)	HbA1c > 8.0% (N = 417)	p
Mean HbA1c level	6.2 ± 0.3	6.8 ± 0.1	7.3 ± 0.1	7.7 ± 0.1	9.0 ± 0.8	< 0.001
Number of HbA1c measurement	5.0 [3.0, 7.0]	4.0 [3.0, 6.0]	5.0 [3.0, 6.0]	4.0 [3.0, 6.0]	4.0 [3.0, 6.0]	0.05
Hb A1c measurement interval, days	447 [272, 636]	446 [340, 674]	468 [351, 671]	512 [368, 726]	467 [344, 653]	0.055
Mean follow-up period, years	6.3 ± 2.3	6.5 ± 2.3	6.6 ± 2.3	6.3 ± 2.2	6.4 ± 2.5	0.798
Age (years)	62.9 ± 10.8	60.9 ± 11.0	61.4 ± 11.2	59.5 ± 11.2	58.0 ± 11.2	< 0.001
Age ≥ 65 (%)	113 (46.1)	108 (39.6)	109 (44.9)	72 (35.0)	121 (29.0)	< 0.001
Male (%)	179 (73.1)	211 (77.3)	185 (76.1)	148 (71.8)	289 (69.3)	0.147
BMI (kg/m^2^)	24.8 ± 3.2	25.1 ± 3.1	25.2 ± 3.3	24.9 ± 3.2	24.7 ± 3.4	0.3
Diagnosis (%)						0.24
STEMI	128 (52.2)	127 (46.5)	122 (50.2)	104 (50.5)	184 (44.1)	
NSTEMI	117 (47.8)	146 (53.5)	121 (49.8)	102 (49.5)	233 (55.9)	
Cardiac arrest on arrival (%)	2 (0.8)	2 (0.7)	0 (0.0)	1 (0.5)	1 (0.2)	0.553
Systolic BP (mmHg)	130.9 ± 26.5	134.2 ± 27.1	131.5 ± 26.5	132.7 ± 27.1	130.4 ± 28.5	0.45
Diastolic BP (mmHg)	79.7 ± 16.0	80.5 ± 15.6	79.9 ± 17.2	80.2 ± 17.4	78.9 ± 17.0	0.749
Heart rate (bpm)	79.1 ± 20.8	79.4 ± 18.5	80.2 ± 17.4	81.4 ± 18.0	82.2 ± 19.8	0.223
Killip class ≥ 3 (%)	223 (91.0)	252 (92.3)	225 (92.6)	182 (88.3)	362 (86.8)	0.063
Hypertension (%)	168 (68.6)	157 (57.5)	143 (58.8)	132 (64.1)	228 (54.7)	0.005
Dyslipidemia on drug treatment (%)	40 (16.3)	53 (19.4)	42 (17.3)	39 (18.9)	70 (16.8)	0.853
Current smoker (%)	89 (36.3)	115 (42.1)	95 (39.1)	76 (36.9)	187 (44.8)	0.157
History of MI (%)	7 (2.9)	4 (1.5)	10 (4.1)	8 (3.9)	14 (3.4)	0.432
History of PCI (%)	17 (6.9)	15 (5.5)	13 (5.3)	16 (7.8)	20 (4.8)	0.572
History of stroke (%)	23 (9.4)	24 (8.8)	14 (5.8)	6 (2.9)	25 (6.0)	0.037
History of cancer (%)	8 (3.3)	10 (3.7)	4 (1.6)	7 (3.4)	9 (2.2)	0.545
LV EF, %	53.4 ± 11.0	53.3 ± 11.0	54.4 ± 10.4	53.4 ± 11.0	51.3 ± 10.5	0.003
Total cholesterol (mg/dL)	169.5 ± 41.2	173.7 ± 41.9	176.4 ± 42.2	179.5 ± 45.3	182.0 ± 46.6	0.008
Triglyceride (mg/dL)	129.9 ± 104.5	141.6 ± 96.5	153.2 ± 118.6	156.1 ± 113.3	161.1 ± 141.2	0.013
HDL cholesterol (mg/dL)	40.3 ± 10.3	39.8 ± 10.4	39.6 ± 11.6	39.5 ± 8.9	39.4 ± 9.4	0.866
LDL cholesterol (mg/dL)	105.3 ± 35.6	108.9 ± 36.3	108.6 ± 35.2	112.1 ± 39.8	113.5 ± 39.2	0.09
eGFR (mL/min/1.73 m^2^)	73.3 [55.1, 91.9]	81.6 [63.1, 95.8]	81.0 [60.3, 97.1]	78.5 [61.4, 97.8]	79.9 [59.2, 99.9]	0.015
Dialysis (%)	8 (3.3)	4 (1.5)	3 (1.2)	1 (0.5)	9 (2.2)	0.237
eGFR ≤ 30 (%)	21 (8.6)	18 (6.6)	18 (7.4)	9 (4.4)	27 (6.5)	0.495
30 < eGFR ≤ 60 (%)	53 (21.6)	38 (13.9)	42 (17.3)	38 (18.4)	77 (18.5)	0.244
GRACE score	139.0 [116.0, 156.0]	133.0 [112.0, 153.0]	135.0 [107.5, 158.0]	126.0 [109.0, 150.8]	133.0 [112.0, 153.0]	0.251
GNRI	97.8 ± 8.6	99.7 ± 16.9	97.9 ± 7.7	97.4 ± 8.2	96.3 ± 8.1	0.002^a^
Discharge medication						
Aspirin (%)	244 (99.6)	266 (97.4)	239 (98.4)	205 (99.5)	412 (98.8)	0.204
Clopidogrel (%)	213 (86.9)	237 (86.8)	215 (88.5)	181 (87.9)	365 (87.5)	0.98
Potent P2Y12 inhibitor (%)	31 (12.7)	36 (13.2)	28 (11.5)	25 (12.1)	52 (12.5)	0.986
Statin (%)	219 (94.4)	239 (96.8)	216 (96.4)	181 (94.3)	375 (96.6)	0.437
Beta blocker (%)	196 (92.9)	238 (96.0)	209 (94.1)	179 (95.7)	334 (93.3)	0.487
RAS blocker (%)	161 (65.7)	182 (66.7)	167 (68.7)	138 (67.0)	305 (73.1)	0.227
Anticoagulation (%)	6 (2.4)	9 (3.3)	8 (3.3)	7 (3.4)	11 (2.6)	0.95
Glucose-lowering treatment (%)	128 (52.2%)	172 (63.0%)	144 (59.3%)	143 (69.4%)	294 (70.5%)	< 0.001
Insulin (%)	11 (4.5)	19 (7.0)	17 (7.0)	21 (10.2)	57 (13.8)	< 0.001
Metformin (%)	76 (31.1)	117 (42.9)	95 (39.3)	105 (51.5)	193 (46.6)	< 0.001
Sulfonylurea (%)	51 (20.8)	74 (27.2)	68 (28.1)	75 (36.8)	160 (38.6)	< 0.001
Thiazolidinedione (%)	0 (0.0)	0 (0.0)	0 (0.0)	1 (0.5)	2 (0.5)	0.498
DPP4 inhibitor (%)	10 (4.1)	17 (6.3)	16 (6.6)	13 (6.3)	28 (6.8)	0.699
α-glucosidase (%)	8 (3.3)	14 (5.2)	14 (5.8)	17 (8.3)	33 (8.0)	0.097
Angiographic data						
Culprit lesion (%)						0.466
LM/LAD	128 (52.3)	130 (47.6)	116 (47.7)	106 (51.5)	196 (47.0)	
RCA/LCX	117 (47.8)	143 (52.4)	127 (52.3)	100 (48.6)	221 (53.0)	
LM disease (%)	21 (8.6)	11 (4.0)	13 (5.3)	13 (6.3)	24 (5.8)	0.281
Multivessel disease (%)	96 (39.2)	106 (38.8)	87 (35.8)	84 (40.8)	157 (37.6)	0.85
Complete revascularization (%)	179 (73.1)	183 (67.0)	163 (67.1)	146 (70.9)	292 (70.0)	0.531
Total stent number	1.7 ± 0.9	1.6 ± 0.9	1.7 ± 0.8	1.6 ± 1.0	1.7 ± 1.0	0.299
Mean stent diameter	3.2 ± 0.4	3.2 ± 0.4	3.1 ± 0.4	3.1 ± 0.4	3.1 ± 0.4	0.071
Total stent length	34.9 ± 22.8	34.4 ± 20.5	33.9 ± 18.4	35.7 ± 22.5	37.0 ± 23.1	0.387

Values are reported as n (%), mean ± SD, or median [interquartile range]

*HbA1c* glycated hemoglobin A, *BMI* body mass index, *STEMI* ST-elevation myocardial infarction, *NSTEMI* non-ST-elevation myocardial infarction, *BP* blood pressure, *MI* myocardial infarction, *PCI* percutaneous coronary intervention, *CABG* coronary artery bypass surgery, *LV EF* left ventricular ejection fraction, *eGFR* estimated glomerular filtration rate, *HDL* high-density lipoprotein, *LDL* low-density lipoprotein, *GRACE* Global Registry of Acute Coronary Events, *GNRI* Geriatric Nutritional Risk Index, *RAS* renin angiotensin system blocker, *PPAR* peroxisome proliferator-activated receptor, *DPP4* dipeptidyl peptidase-4, *LM* left main, *LAD* left anterior descending artery, *RCA* right coronary artery, *LCX* left circumflex artery

^a^In Bonferroni post hoc analysis, only GNRI of patients with HbA1c > 8.0% was significantly lower than that of patients with HbA1c of 6.5 to 7.0%

### Clinical outcomes

**Fig. 2:**
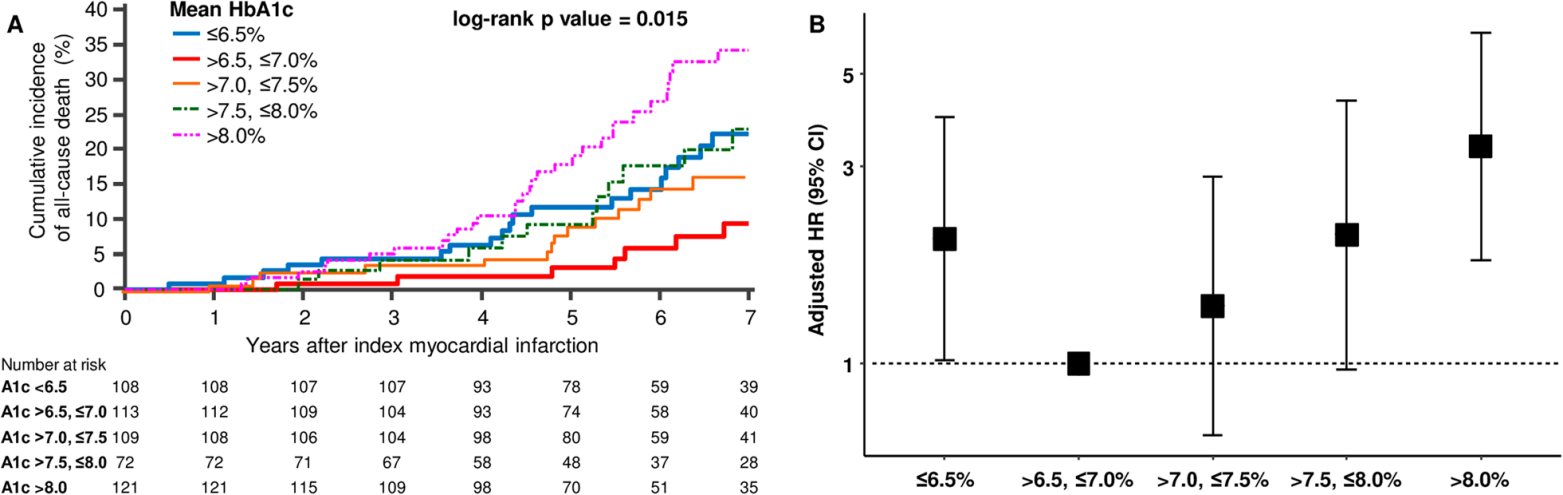
Incidence and adjusted hazard ratio of all-cause mortality according to HbA1c group. **A** Kaplan–Meier curve of all-cause mortality according to HbA1c group. **B** Hazard ratio and 95% confidence interval by multivariate Cox regression (Model 3: adjustment with age, sex, hypertension, current smoke, chronic kidney disease, previous stroke, the type of myocardial infarction, left ventricular ejection fraction, GRACE score, and complete revascularization) according to HbA1c group. *HR* hazard ratio, *CI* confidence interval

All-cause mortality occurred in 158 (11.4%) patients. The relationship between HbA1C and the incidence of all-cause mortality followed a J-shaped curve, with increased incidence of all-cause mortality at low and high HbA1c (Table 2, Fig. 2). Patients with a mean HbA1c of 6.5% to 7.0% had the lowest all-cause mortality. Cubic regression spline analysis revealed that the adjusted risk of all-cause mortality was the lowest for those with a HbA1c of 6.7% and tended to increase in those with lower HbA1c and higher HbA1c, describing J-curve pattern (Additional file 1: Fig. S1). Compared with the reference group (HbA1c of 6.5% to 7.0%), the risk of mortality increased twofold in patients with HbA1c ≤ 6.5% and by more than threefold in those with HbA1c > 8.0% after adjustment for covariates of Model 3 (Table 3, Fig. 2).

**Table 2:** Clinical outcomes of the patients according to the glycated hemoglobin level

	HbA1c ≤ 6.5%	6.5% < HbA1c ≤ 7.0%	7.0% < HbA1c ≤ 7.5%	7.5% < HbA1c ≤ 8.0%	HbA1c > 8.0%	p
All cause death (%)	29 (51.2)	18 (28)	25 (39.9)	20 (26.1)	66 (176.3)	0.004
Cardiovascular death (%)	15 (26.5)	11 (17.1)	15 (23.9)	14 (18.3)	56 (149.6)	< 0.001
Non-cardiovascular death (%)	14 (24.7)	7 (10.9)	10 (16)	6 (7.8)	10 (26.7)	0.172
Myocardial infarction (%)	11 (19.4)	15 (23.3)	17 (27.1)	14 (18.3)	36 (96.2)	0.281
Stroke (%)	12 (21.2)	12 (18.7)	11 (17.6)	10 (13)	13 (34.7)	0.768
Composite of cardiovascular death, myocardial infarction, and stroke	34 (60)	39 (60.6)	36 (57.5)	32 (41.7)	90 (240.4)	0.033

Values are reported as n (incidence rate per 1000 person-years)

*HbA1c* glycated hemoglobin A

**Table 3:** Hazard ratios and 95% CIs of mortality according to the glycated hemoglobin level

HbA1c group	Unadjusted HR			Model 1			Model 2			Model 3		
	HR	95% CI	p value	HR	95% CI	p value	HR	95% CI	p value	HR	95% CI	p value
6.5% < HbA1c ≤ 7.0%	Ref			Ref			Ref			Ref		
HbA1c ≤ 6.5%	2.17	1.11–4.24	0.024	2.16	1.1–4.24	0.025	2.09	1.02–3.88	0.043	2.00	1.02–3.95	0.045
7.0% < HbA1c ≤ 7.5%	1.61	0.8–3.27	0.183	1.68	0.83–3.41	0.150	1.67	0.82–3.39	0.156	1.38	0.67–2.82	0.381
7.5% < HbA1c ≤ 8.0%	1.65	0.79–3.43	0.181	2.01	0.96–4.21	0.064	2.11	1–4.43	0.049	2.05	0.97–4.33	0.059
HbA1c > 8.0%	2.64	1.44–4.85	0.002	3.60	1.93–6.72	< 0.001	3.51	1.88–6.56	< 0.001	3.35	1.78–6.29	< 0.001

Model 1: Hazard ratios categorized HbA1c adjusted by age, sex, and body mass index. Model 2: Hazard ratios categorized HbA1c adjusted by model 1 plus systolic blood pressure, hypertension, current smoke, chronic kidney disease, and previous stroke. Model 3: Hazard ratios categorized HbA1c adjusted by model 2 plus the type of myocardial infarction, left ventricular ejection fraction, low-density lipoprotein cholesterol, GRACE score, and complete revascularization. The C-index of models was 0.764 (95% confidence interval [CI] 0.723–0.805), 0.800 (95% CI 0.764–0.837), and 0.825 (95% CI 0.791–0.859) for models 1, 2, and 3, respectively

*HbA1c* glycated hemoglobin A, *HR* hazard ratio, *CI* confidence interval, *Ref*. reference

For the secondary outcomes, cardiovascular death occurred in 111 (8.0%) patients, 93 (6.7%) patients experienced myocardial infarction, and 58 (4.2%) experienced ischemic stroke. Cardiovascular death was increased from in patients with HbA1c > 7.5% and ≤ 8.0% and significantly increased in those with HbA1c > 8.0% (adjusted HR 4.24, 95% CI 2.17–8.27, p < 0.001), but not significantly increased in patients with HbA1c ≤ 6.5% (adjusted HR 1.44, 95% CI 0.65–3.15, p = 0.368) compared to patients with HbA1c of 6.5% to 7.0% after full adjustment (Additional file 1: Table S1). There was no significant difference in the rates of myocardial infarction and stroke among groups (Table 2). The risk of the composite of cardiovascular death, myocardial infarction, and stroke was significantly increased only in patients with HbA1c > 8.0% (adjusted HR 1.59, 95% CI 1.08–2.34, p = 0.018) (Additional file 1: Table S2).

### Subgroup analysis of the relationship between HbA1c and mortality according to age

**Fig. 3:**
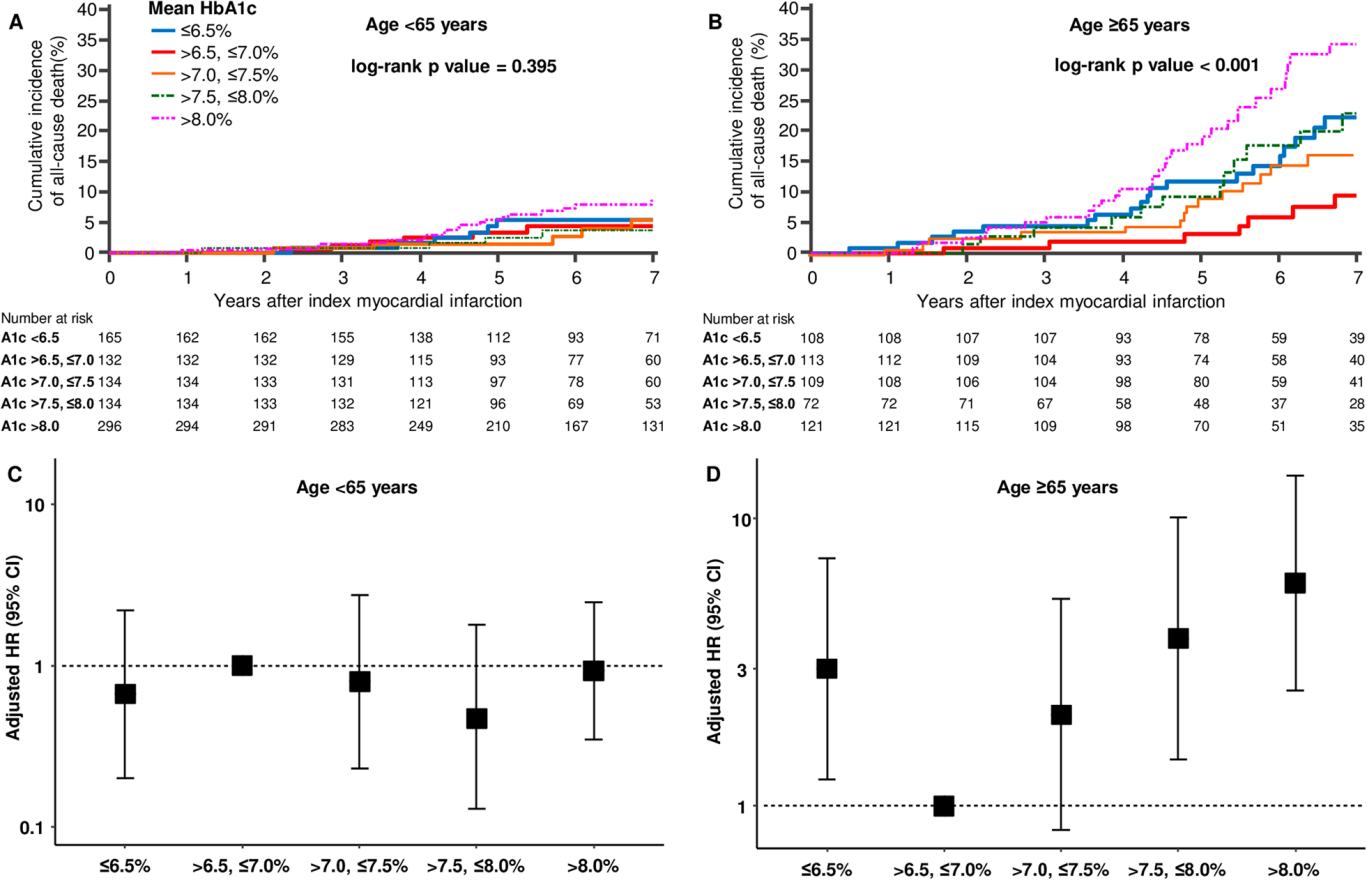
Subgroup analysis of the primary outcome by the age of 65 years. **A** Kaplan–Meier curve of all-cause mortality in patients younger than 65 years old according to HbA1c group. **B** Kaplan–Meier curve of all-cause mortality in patients 65 years or older according to HbA1c group. **C** Hazard ratio and 95% confidence interval in patients younger than 65 years according to HbA1c group. **D** Hazard ratio and 95% confidence interval in patients 65 years or older according to HbA1c group. *HR* hazard ratio, *CI* confidence interval

When the patients were divided by 65 years, a bimodal relationship between HbA1c and all-cause mortality was not observed in patients aged below 65 years but in patients aged 65 years or older. The incidence of mortality was not different in patients below the age of 65 years among HbA1c groups. In contrast, the increase of hazard rates of mortality at low and high HbA1c remained significant after full adjustment in patients aged 65 years or older. However, the interaction between age and HbA1c was not significant (p = 0.685). The risk of mortality increased threefold in the group with HbA1c ≤ 6.5% and sixfold with HbA1c > 8.0% in patients aged 65 years or older (Fig. 3). Patients aged 65 years or older were more likely to have lower HbA1c, low body mass index, diagnosis of NSTEMI, history of stroke, lower left ventricular function, lower cholesterol level, worse kidney function, complex coronary disease, and a lower rate of complete revascularization compared to those less than 65 years of age (Additional file 1: Table S3).

### Sensitivity analysis to exclude reverse causality

For the possibility that the higher risks of all-cause deaths in patients with low mean HbA1c levels may be due to reverse causality, we performed sensitivity analyses excluding from primary analyses those individuals who died in the first 1 year after the last HbA1c measurement. Even after excluding 101 patients, the baseline characteristics of patients and the trend of J-curve relationship between the HbA1c and all-cause mortality were maintained in the sensitivity analyses (Additional file 1: Tables S4–S6).

## Discussion

The mean HbA1c level during follow-up of 1348 diabetic patients who underwent PCI for AMI showed a bimodal (J-curve) relationship with all-cause mortality. Compared to patients with HbA1c in the range of 6.5% to 7.0% who had the lowest risk of mortality, the risk of mortality was significantly higher in patients with HbA1c ≤ 6.5% and those with HbA1c > 8.0% after adjusting for potential confounding factors. The increased mortality in patients with HbA1c < 6.5% was driven by both cardiovascular death and non-cardiovascular death, whereas excess mortality in patients with HbA1c > 8.0% was attributed to high cardiovascular death. Further, the J-curve relationship was more robust in elderly patients (age ≥ 65 years) than younger patients (age < 65 years).

Glycemic control is fundamental to diabetes management. Meanwhile, the influence of intensive glycemic control on macrovascular complications (myocardial infarction, stroke, and peripheral artery disease) and mortality is disputed. Large randomized controlled trials investigating intensive versus standard glycemic control and vascular outcomes did not show any benefit of intensive glycemic control [15, 16]. A single study found that intensive control increased mortality [5]. The randomized controlled trials did not enroll patients in the acute phase of CV disease but only patients with a history of CV disease. The association between glycemic control and CV outcome after AMI, a very high-risk disease, is unclear. Several observational studies were performed to elucidate the relationship between glycemic control and CV outcomes in diabetic patients who underwent PCI. Poor glycemic control was associated with an increased risk of mortality (HbA1c > 10%) [17] and CV events (HbA1c > 7%) [18]. A study reported that stringent glycemic control (HbA1c < 6.5%) was associated with the risk of cardiovascular death or sudden death after PCI [19]. However, the proportion of patients with AMI was 10–20% in those previous studies. The effect of adverse events due to intensive and poor glycemic control on patients with coronary artery disease can be pronounced in patients with AMI. Furthermore, two studies used baseline pre-procedural HbA1c, which partially reflects the glycemic control during the follow-up period after PCI. Our study used mean HbA1c levels during long-term follow-up of AMI patients. We demonstrated that both poor and stringent glycemic control was associated with increased mortality in AMI patients.

It is well known that better glycemic control (target HbA1c < 7%) significantly reduces the rates of development and progression of microvascular (retinopathy, neuropathy, and diabetic kidney disease) complications [20]. Other analyses also suggest that further lowering of HbA1c from 7 to 6% is associated with further reduction in the risk of microvascular complications, although the absolute risk reductions become much smaller [11]. To prevent microvascular complications of diabetes, controlling HbA1c ≤ 6.5% is justified by adequate evidence as a treatment target for patients with a low risk of hypoglycemia. Whereas, subgroup analyses of the VADT study suggest that the potential risks of intensive glycemic control may outweigh its benefits in higher-risk patients [21]. Patients with advanced atherosclerosis or advanced age/frailty may benefit from less aggressive targets [22].

Another characteristic feature of our study was that non-cardiovascular death, as well as cardiovascular death, tended to be high in patients with low mean HbA1c. Other studies have reported higher cancer or non-specific mortality in patients with severe hypoglycemia [13]. There is likely to be a shared underlying cause of both hypoglycemia and mortality, such as diminished physiologic reserve. Since the rate of glucose-lowering treatment was lower in patients with low mean HbA1c in our study, the diminished physiologic reserve in those with low mean HbA1c might have caused high mortality. However, the degree of malnutrition of patients with low mean HbA1c was similar to that of patients with high mean HbA1c. Moreover, we could not observe the reverse causality between HbA1c and all-cause mortality in the sensitivity analysis. The mechanisms directly linking low mean HbA1c to non-cardiovascular deaths are unclear.

Given the substantially increased risk of hypoglycemia in diabetic patients with CV disease and in elderly patients, the risks of lower glycemic targets may outweigh the potential benefits in terms of microvascular complications in these patients. In this study, we compared mortality in two groups categorized by the age of 65 years. The differences in mortality risk according to glycemic control were blunted in the younger patients (age < 65 years). The neutral effect of glycemic control on mortality in younger patients was also observed in the UKPDS 33 study, which showed no difference in CV outcome between intensive glycemic control and diet control in diabetic patients with an average age of 53 years [23]. Stringent glycemic control (HbA1c < 6.5%) in young AMI patients would be safe and necessary to prevent long-term microvascular and macrovascular complications [24]. Conversely, the association between low and high mean HbA1c and increased all-cause mortality was prominent in elderly patients (age ≥ 65 years). A recent study of the GERODIAB trial has reported that lower mean HbA1c was associated with lower mortality in diabetic patients aged 70 years and older [25]. However, a more extensive registry study enrolled 27,965 patients older than 50 years and showed a similar J-curve relationship between mean HbA1c and mortality [26]. Another observational study involving 71,092 patients older than 60 years old also found a J-curve relationship between HbA1c and mortality [27]. The ACCORD trial comparing glycemic control in diabetic patients with a mean age of 62 years was halted early due to increased mortality rate in the intensive (HbA1c < 6%) compared with the standard treatment arm (HbA1c 7.0–7.9%) with a similar increase in cardiovascular deaths [2]. Severe hypoglycemia was significantly more likely in patients under intensive glycemic control. Our study suggested that glycemic control targeting HbA1c of 6.5–7.0% would be ideal for preventing future complications in elderly patients, consistent with the current practice guideline [9, 11]. The mortality findings and the relatively intense efforts required to achieve near euglycemia should be considered when setting glycemic targets in elderly patients. Since we demonstrated a differential effect of intensive glycemic control on mortality according to age, glycemic control after AMI should be individualized according to age or the presence of multiple comorbidities. Further studies are needed under detailed clinical settings.

HbA1c is the metric used to date in clinical trials demonstrating the benefits of improved glycemic control and has an excellent correlation with the mean glucose level. However, the HbA1c test is an indirect measure of average glycemia and is subject to limitations. Conditions such as recent blood transfusion and end-stage kidney disease may result in discrepancies between the HbA1c result and the patient’s true mean glycemia [11]. In patients with AMI, clinically relevant bleeding requiring transfusion is frequent within the 1st year after PCI. We used more than three measurements with an interval of 1.3 years to minimize the effect of possible transfusion on the HbA1c level early after PCI. Our cohort included a few patients undergoing dialysis, which was not different among the HbA1c groups.

This study has several limitations. First, as an observational study, we cannot conclude that the observed associations were causal. Unmeasured confounders may affect our results. Despite a relatively small number of patients enrolled, we adjusted with known confounding factors for the mortality after AMI. Second, new hypoglycemic agents such as glucagon-like peptide 1 receptor agonist and sodium-glucose cotransporter 2 inhibitors were not prescribed for our patient population at discharge. We enrolled patients who underwent PCI until the year of 2014, when those medications were not available. Treatment with new hypoglycemic agents lowered HbA1c by 0.4–0.5% of HbA1c and demonstrated the mortality benefit in recent randomized trials [28, 29]. Further studies are required to assess whether the J-Curve relationship between HbA1c and mortality was maintained after using new hypoglycemic agents. Third, we analyzed 1384 patients from 4093 diabetic patients, raising concerns of possible selection bias and reducing study generalizability. Baseline characteristics between included and excluded diabetic patients were compared in the Additional file 1: Table S7. Excluded patients had higher risk characteristics such as older ages, higher Killip class, more co-morbidities, and lower left ventricular systolic function. Excluded patients are those at a higher risk of death than included patients. The shorter follow-up period of excluded patients may not be suitable for examining the effect of long-term glycemic control in this study. Fourth, HbA1c was measured irregularly and with wide intervals. However, the measurement interval was not significantly different among the HbA1c groups. The mean HbA1c level of our study reflects the values over a median of 3 years. Our study may represent the association between long-term glycemic control and long-term mortality in AMI patients with diabetes.

## Conclusions

Mean HbA1c levels was associated with a J-curve relationship with all-cause mortality during long-term follow-up of diabetic patients with AMI. The mortality rate was the lowest in patients with HbA1C of 6.5–7.0% and increased when HbA1c was 6.5% or less and higher than 8.0%. The J-curve relationship between mean HbA1c and mortality was more robust in elderly patients.

## Supplementary Information

**Additional file 1: Table S1.** Hazard ratios and 95% CIs of cardiovascular mortality according to the glycated hemoglobin level. **Table S2.** Hazard ratios and 95% CIs of the composite of cardiovascular mortality, myocardial infarction, and stroke according to the glycated hemoglobin level. **Table S3.** Baseline characteristics of the patients according to age. **Table S4.** Baseline characteristics of the patients according to the glycated hemoglobin level after excluding individuals who died in the first 1 years after the last HbA1c measurement. **Table S5.** Hazard ratios and 95% CIs of mortality according to the glycated hemoglobin level in patients after excluding individuals who died in the first 1 year after the last HbA1c measurement. **Table S6.** Hazard ratios and 95% CIs of mortality according to the glycated hemoglobin level in patients after exclusion according to age. **Table S7.** Baseline characteristics of diabetic patients who were excluded from this study and included in this study. **Figure S1.** Cubic spline curve showing the hazard of all-cause mortality according to mean HbA1c.

## Data Availability

The data of this study may be available on reasonable request to the corresponding author.

## Abbreviations

AMI: Acute myocardial infarction
HbA1c: Glycated hemoglobin
HR: Hazard ratio
CI: Confidence interval
CV: Cardiovascular
STEMI: ST-segment elevation myocardial infarction
NSTEMI: Non-ST-segment elevation myocardial infarction
PCI: Percutaneous coronary intervention
GRACE: Global registry of acute coronary events
