# Supplementary material for: J-curve relationship between long term glycemic control and mortality in diabetic patients with acute myocardial infarction undergoing percutaneous coronary intervention
Source: Cardiovasc Diabetol. 2021 Dec 15;20:234. doi: 10.1186/s12933-021-01428-x (PMC8675510; doi:10.1186/s12933-021-01428-x)
Supplement: Supplementary file 1 — Additional file 1: Table S1. Hazard ratios and 95% CIs of cardiovascular mortality according to the glycated hemoglobin level. Table S2. Hazard ratios and 95% CIs of the composite of cardiovascular mortality, myocardial infarction, and stroke according to the glycated hemoglobin level. Table S3. Baseline characteristics of the patients according to age. Table S4. Baseline characteristics of the patients according to the glycated hemoglobin level after excluding individuals who died in the first 1 years after the last HbA1c measurement. Table S5. Hazard ratios and 95% CIs of mortality according to the glycated hemoglobin level in patients after excluding individuals who died in the first 1 year after the last HbA1c measurement. Table S6. Hazard ratios and 95% CIs of mortality according to the glycated hemoglobin level in patients after exclusion according to age. Table S7. Baseline characteristics of diabetic patients who were excluded from this study and included in this study. Figure S1. Cubic spline curve showing the hazard of all-cause mortality according to mean HbA1c. [file 12933_2021_1428_MOESM1_ESM.docx]

**Additional data**

**Table S1.** **Hazard Ratios and 95% CIs of cardiovascular mortality according to the glycated hemoglobin level**

| HbA1c group | **Unadjusted HR** | | | **Model 1** | | | **Model 2** | | | **Model 3** | | |  |
| --- | --- | --- | --- | --- | --- | --- | --- | --- | --- | --- | --- | --- | --- |
|  | HR | 95% CI | p value | HR | 95% CI | p value | HR | 95% CI | p value | HR | 95% CI | p value | |
| **6.5% <HbA1c ≤7.0%** | Ref. |  |  | Ref. |  |  | Ref. |  |  | Ref. |  |  | |
| **HbA1c ≤6.5%** | 1.57 | 0.72-3.41 | 0.257 | 1.57 | 0.72-3.44 | 0.254 | 1.38 | 0.63-3.03 | 0.420 | 1.44 | 0.65-3.15 | 0.368 | |
| **7.0% <HbA1c ≤7.5%** | 1.48 | 0.68-3.23 | 0.321 | 1.60 | 0.74-3.5 | 0.235 | 1.59 | 0.73-3.48 | 0.241 | 1.19 | 0.54-2.63 | 0.669 | |
| **7.5% <HbA1c ≤8.0%** | 1.76 | 0.8-3.88 | 0.161 | 2.14 | 0.96-4.74 | 0.063 | 2.28 | 1.02-5.09 | 0.044 | 2.29 | 1.03-5.13 | 0.043 | |
| **HbA1c >8.0%** | 3.35 | 1.76-6.4 | <0.001 | 4.57 | 2.36-8.87 | <0.001 | 4.60 | 2.37-8.94 | <0.001 | 4.24 | 2.17-8.27 | <0.001 | |

Model 1: Hazard ratios categorized HbA1c adjusted by age, sex, and body mass index. Model 2: Hazard ratios categorized HbA1c adjusted by model 1 plus systolic blood pressure, hypertension, current smoke, chronic kidney disease, and previous stroke. Model 3: Hazard ratios categorized HbA1c adjusted by model 2 plus the type of myocardial infarction, left ventricular ejection fraction, low-density lipoprotein cholesterol, GRACE score, and complete revascularization. HbA1c, glycated hemoglobin A; HR, hazard ratio; CI, confidence interval; Ref., reference

**Table S2. Hazard Ratios and 95% CIs of the composite of cardiovascular mortality, myocardial infarction, and stroke according to the glycated hemoglobin level**

| HbA1c group | **Unadjusted HR** | | | **Model 1** | | | **Model 2** | | | **Model 3** | | |  |
| --- | --- | --- | --- | --- | --- | --- | --- | --- | --- | --- | --- | --- | --- |
|  | HR | 95% CI | p value | HR | 95% CI | p value | HR | 95% CI | p value | HR | 95% CI | p value | |
| **6.5% <HbA1c ≤7.0%** | Ref. |  |  | Ref. |  |  | Ref. |  |  | Ref. |  |  | |
| **HbA1c ≤6.5%** | 0.97 | 0.61-1.54 | 0.907 | 0.92 | 0.58-1.46 | 0.727 | 0.86 | 0.54-1.37 | 0.539 | 0.87 | 0.55-1.38 | 0.555 | |
| **7.0% <HbA1c ≤7.5%** | 1.01 | 0.64-1.59 | 0.958 | 1.03 | 0.65-1.62 | 0.909 | 1.02 | 0.65-1.6 | 0.946 | 1.00 | 0.64-1.58 | 0.989 | |
| **7.5% <HbA1c ≤8.0%** | 1.11 | 0.7-1.77 | 0.663 | 1.16 | 0.73-1.86 | 0.525 | 1.18 | 0.73-1.89 | 0.500 | 1.20 | 0.75-1.92 | 0.453 | |
| **HbA1c >8.0%** | 1.51 | 1.04-2.2 | 0.031 | 1.67 | 1.14-2.45 | 0.008 | 1.65 | 1.12-2.41 | 0.011 | 1.59 | 1.08-2.34 | 0.018 | |

Model 1: Hazard ratios categorized HbA1c adjusted by age, sex, and body mass index. Model 2: Hazard ratios categorized HbA1c adjusted by model 1 plus systolic blood pressure, hypertension, current smoke, chronic kidney disease, and previous stroke. Model 3: Hazard ratios categorized HbA1c adjusted by model 2 plus the type of myocardial infarction, left ventricular ejection fraction, low-density lipoprotein cholesterol, GRACE score, and complete revascularization. HbA1c, glycated hemoglobin A; HR, hazard ratio; CI, confidence interval; Ref., reference

**Table S3. Baseline characteristics of the patients according to age**

|  | **Age <65 years**  **(N= 861)** | **Age ≥ 65 years**  **(N= 523)** | **p** |
| --- | --- | --- | --- |
| Mean HbA1c level | 7.7 ± 1.2 | 7.4 ± 1.1 | <0.001 |
| Number of HbA1c measurement | 5.0 [3.0, 6.0] | 4.0 [3.0, 6.0] | 0.002 |
| Hb A1c measurement interval, days | 471 [ 346, 670] | 467 [338, 670] | 0.609 |
| Mean follow-up period, years | 6.5 ± 2.3 | 6.2 ± 2.3 | 0.005 |
| Age (years) | 53.2 ± 7.4 | 71.9 ± 5.1 | <0.001 |
| Male (%) | 725 (84.2) | 287 (54.9) | <0.001 |
| BMI (kg/m^2^) | 25.3 ± 3.3 | 24.2 ± 3.1 | <0.001 |
| Diagnosis (%) |  |  | 0.003 |
| STEMI | 475 (55.2) | 244 (46.7) | 0.003 |
| NSTEMI | 386 (44.8) | 279 (53.3) |  |
| Cardiac arrest on arrival (%) | 4 (0.5) | 2 (0.4) | 1 |
| Systolic BP (mmHg) | 132.1 ± 26.7 | 131.3 ± 28.2 | 0.626 |
| Diastolic BP (mmHg) | 81.3 ± 16.5 | 77.1 ± 16.5 | <0.001 |
| Heart rate (bpm) | 80.5 ± 18.3 | 80.9 ± 20.3 | 0.671 |
| Killip class (%) |  |  | 0.039 |
| 1 | 736 (85.5) | 426 (81.5) |  |
| 2 | 47 (5.5) | 35 (6.7) |  |
| 3 | 33 (3.8) | 37 (7.1) |  |
| 4 | 45 (5.2) | 25 (4.8) |  |
| Hypertension (%) | 433 (50.3) | 395 (75.5) |  |
| Dyslipidemia (%) | 163 (18.9) | 81 (15.5) | 0.119 |
| Current smoker (%) | 470 (54.6) | 92 (17.6) | <0.001 |
| History of MI (%) | 25 (2.9) | 18 (3.4) | 0.689 |
| History of PCI (%) | 43 (5.0) | 38 (7.3) | 0.104 |
| History of stroke (%) | 35 (4.1) | 57 (10.9) | <0.001 |
| Cancer (%) | 15 (1.7) | 23 (4.4) | 0.006 |
| LV EF, % | 53.7 ± 10.5 | 51.7 ± 11.2 | 0.001 |
| Total cholesterol (mg/dL) | 182.9 ± 44.4 | 166.6 ± 41.3 | <0.001 |
| Triglyceride (mg/dL) | 168.0 ± 136.7 | 118.9 ± 72.7 | <0.001 |
| HDL cholesterol (mg/dL) | 39.5 ± 9.4 | 39.9 ± 11.1 | 0.461 |
| LDL cholesterol (mg/dL) | 114.0 ± 38.1 | 103.3 ± 35.4 | <0.001 |
| eGFR (ml/min/1.73m2) | 84.9 ± 23.8 | 61.5 ± 23.6 | <0.001 |
| Dialysis (%) | 14 (1.6) | 11 (2.1) | 0.661 |
| eGFR ≤ 30 (%) | 29 (3.4) | 64 (12.2) | <0.001 |
| 30 <eGFR≤ 60 (%) | 81 (9.4) | 167 (31.9) | <0.001 |
| GRACE score | 124.1 ± 31.6 | 154.9 ± 32.1 | <0.001 |
| GNRI | 99.1 ± 11.8 | 95.4 ± 7.5 | <0.001 |
| **Discharge medication** |  |  |  |
| Aspirin (%) | 852 (99.0) | 514 (98.3) | 0.406 |
| Clopidogrel (%) | 732 (85.0) | 479 (91.6) | <0.001 |
| Potent P2Y12 inhibitor (%) | 129 (15.0) | 43 (8.2) | <0.001 |
| Statin (%) | 771 (96.5) | 459 (94.8) | 0.192 |
| Beta blocker (%) | 739 (95.2) | 417 (92.7) | 0.082 |
| RAS blocker (%) | 617 (71.7) | 336 (64.2) | 0.004 |
| Anticoagulation (%) | 21 (2.4) | 20 (3.8) | 0.19 |
| Hypoglycemic treatment (%) | 537 (62.4%) | 344 (65.8%) | 0.205 |
| Insulin (%) | 73 (8.5) | 52 (10.0) | 0.392 |
| Metformin (%) | 385 (44.9) | 201 (38.7) | 0.029 |
| Sulfonylurea (%) | 254 (29.6) | 174 (33.5) | 0.139 |
| Thiazolidinedione (%) | 3 (0.3) | 0 (0.0) | 0.295 |
| DPP4 inhibitor (%) | 53 (6.2) | 31 (6.0) | 0.989 |
| α-glucosidase (%) | 40 (4.7) | 46 (8.9) | 0.003 |
| **Angiographic data** |  |  |  |
| Culprit lesion (%) |  |  | 0.03 |
| LM | 391 (45.4) | 250 (47.8) | 0.03 |
| LAD | 153 (17.8) | 73 (14.0) |  |
| LCX | 15 (1.7) | 20 (3.8) |  |
| RCA | 302 (35.1) | 180 (34.4) |  |
| LM disease (%) | 40 (4.6) | 42 (8.0) | 0.014 |
| Multivessel disease (%) | 489 (56.8) | 365 (69.8) | <0.001 |
| Complete revascularization (%) | 617 (71.7) | 346 (66.2) | 0.036 |
| Total stent number | 1.6 ± 0.9 | 1.7 ± 0.9 | 0.028 |
| Mean stent diameter | 3.2 ± 0.4 | 3.1 ± 0.4 | <0.001 |
| Total stent length | 34.7 ± 21.8 | 36.5 ± 21.5 | 0.139 |

Values are reported as n (%), mean ± SD, or median [interquartile range]. HbA1c, glycated hemoglobin A; BMI, body mass index; STEMI, ST-elevation myocardial infarction; NSTEMI, Non-ST-elevation myocardial infarction; BP, blood pressure; MI, myocardial infarction; PCI, percutaneous coronary intervention; CABG, coronary artery bypass surgery; LV EF, left ventricular ejection fraction; eGFR, estimated glomerular filtration rate; HDL, high-density lipoprotein; LDL, low-density lipoprotein; GRACE, Global Registry of Acute Coronary Events; GNRI, Geriatric Nutritional Risk Index; RAS, renin angiotensin system blocker; PPAR, peroxisome proliferator-activated receptor; DPP4, Dipeptidyl Peptidase-4; LM, left main; LAD, left anterior descending artery; LCX, left circumflex artery; RCA, right coronary artery

**Table S4. Baseline characteristics of the patients according to the glycated hemoglobin level after excluding individuals who died in the first 1 years after the last HbA1c measurement**

|  | **HbA1c ≤6.5%**  **(N= 229)** | **6.5% <HbA1c ≤ 7.0%**  **(N = 259)** | **7.0% <HbA1c ≤ 7.5%**  **(N = 231)** | **7.5% <HbA1c ≤ 8.0%**  **(N = 193)** | **HbA1c > 8.0%**  **(N = 371)** | **p** |
| --- | --- | --- | --- | --- | --- | --- |
| Mean HbA1c level | 6.2 ± 0.3 | 6.8 ± 0.1 | 7.3 ± 0.1 | 7.7 ± 0.1 | 9.0 ± 0.8 | <0.001 |
| Number of HbA1c measurement | 5.7 ± 3.2 | 5.6 ± 3.1 | 5.4 ± 2.8 | 5.0 ± 2.3 | 5.3 ± 2.7 | 0.043 |
| Hb A1c measurement interval, days | 446.8 [271.8, 646.5] | 445.0 [340.4, 672.2] | 477.0 [356.8, 686.5] | 517.7 [373.3, 744.5] | 453.1 [345.7, 657.4] | 0.036 |
| Mean follow-up period, years | 6.5 ± 2.2 | 6.5 ± 2.3 | 6.7 ± 2.3 | 6.5 ± 2.1 | 6.6 ± 2.4 | 0.813 |
| Age (years) | 62.3 ± 10.6 | 60.5 ± 10.7 | 61.1 ± 11.0 | 58.8 ± 10.9 | 57.0 ± 10.9 | <0.001 |
| Age ≥ 65 (%) | 100 (43.7) | 100 (38.6) | 99 (42.9) | 63 (32.6) | 94 (25.3) | <0.001 |
| Male (%) | 168 (73.4) | 201 (77.6) | 178 (77.1) | 140 (72.5) | 263 (70.9) | 0.287 |
| BMI (kg/m^2^) | 25.0 ± 3.1 | 25.2 ± 3.1 | 25.2 ± 3.4 | 25.0 ± 3.2 | 24.8 ± 3.4 | 0.672 |
| Diagnosis (%) |  |  |  |  |  | 0.123 |
| STEMI | 111 (48.5) | 140 (54.1) | 117 (50.6) | 95 (49.2) | 215 (58.0) |  |
| NSTEMI | 118 (51.5) | 119 (45.9) | 114 (49.4) | 98 (50.8) | 156 (42.0) |  |
| Cardiac arrest on arrival (%) | 2 (0.9) | 2 (0.8) | 0 (0) | 1 (0.5) | 1 (0.3) | 0.599 |
| Systolic BP (mmHg) | 130.2 ± 28.6 | 132.7 ± 29.2 | 131.8 ± 26.8 | 132.2 ± 28.2 | 130.2 ± 29.2 | 0.79 |
| Diastolic BP (mmHg) | 79.9 ± 16.1 | 80.3 ± 15.3 | 80.3 ± 17.3 | 80.5 ± 17.2 | 79.2 ± 17.1 | 0.874 |
| Heart rate (bpm) | 79.2 ± 21.2 | 78.8 ± 18.1 | 80.0 ± 17.5 | 81.1 ± 17.5 | 81.4 ± 19.5 | 0.404 |
| Killip class ≥3 (%) | 211 (92.1) | 239 (92.3) | 215 (93.1) | 173 (89.6) | 327 (88.1) | 0.195 |
| Hypertension (%) | 156 (68.1) | 146 (56.4) | 134 (58.0) | 122 (63.2) | 198 (53.4) | 0.005 |
| Dyslipidemia on drug treatment (%) | 38 (16.6) | 50 (19.3) | 38 (16.5) | 36 (18.7) | 66 (17.8) | 0.908 |
| Current smoker (%) | 83 (36.2) | 110 (42.5) | 93 (40.3) | 73 (37.8) | 176 (47.4) | 0.054 |
| History of MI (%) | 6 (2.6) | 4 (1.5) | 8 (3.5) | 8 (4.1) | 10 (2.7) | 0.526 |
| History of PCI (%) | 16 (7.0) | 13 (5.0) | 9 (3.9) | 16 (8.3) | 14 (3.8) | 0.116 |
| History of stroke (%) | 19 (8.3) | 22 (8.5) | 12 (5.2) | 5 (2.6) | 17 (4.6) | 0.029 |
| History of Cancer (%) | 6 (2.6) | 9 (3.5) | 4 (1.7) | 4 (2.1) | 8 (2.2) | 0.762 |
| LV EF, % | 53.4 ± 11.2 | 53.5 ± 10.6 | 54.5 ± 10.4 | 53.9 ± 10.8 | 52.0 ± 10.2 | 0.036 |
| Total cholesterol (mg/dL) | 169.2 ± 41.8 | 174.4 ± 42.0 | 177.4 ± 42.4 | 180.1 ± 46.1 | 182.9 ± 46.2 | 0.005 |
| Triglyceride (mg/dL) | 131.2 ± 105.5 | 142.5 ± 98.0 | 154.9 ± 120.4 | 159.0 ± 115.4 | 164.3 ± 146.4 | 0.012 |
| HDL cholesterol (mg/dL) | 40.1 ± 10.3 | 39.6 ± 10.0 | 39.6 ± 11.7 | 39.3 ± 8.8 | 39.5 ± 9.2 | 0.951 |
| LDL cholesterol (mg/dL) | 105.2 ± 36.1 | 109.7 ± 36.3 | 109.5 ± 35.3 | 112.3 ± 40.5 | 113.8 ± 39.1 | 0.117 |
| eGFR (ml/min/1.73m2) | 77.1 [56.0, 92.7] | 82.0 [63.6, 96.5] | 83.0 [61.5, 97.2] | 82.3 [63.1, 99.7] | 81.2 [62.0, 101.2] | 0.015 |
| Dialysis (%) | 5 (2.2) | 3 (1.2) | 3 (1.3) | 0 (0) | 5 (1.3) | 0.351 |
| eGFR ≤ 30 (%) | 14 (6.1) | 16 (6.2) | 15 (6.5) | 7 (3.6) | 19 (5.1) | 0.695 |
| 30 < eGFR≤ 60 (%) | 52 (22.7) | 35 (13.5) | 37 (16.0) | 31 (16.1) | 60 (16.2) | 0.091 |
| GRACE score | 137.0 [114.0, 154.0] | 133.0 [111.0, 152.5] | 133.0 [106.0, 156.5] | 124.0 [109.0, 149.0] | 130.0 [111.0, 150.0] | 0.209 |
| GNRI | 98.5 ± 8.2 | 100.0 ± 17.3 | 98.1 ± 7.6 | 97.9 ± 8.0 | 97.0 ± 7.9 | 0.055 |
| **Discharge medication** |  |  |  |  |  |  |
| Aspirin (%) | 228 (99.6) | 252 (97.3) | 227 (98.3) | 192 (99.5) | 366 (98.7) | 0.232 |
| Clopidogrel (%) | 198 (86.5) | 223 (86.1) | 204 (88.3) | 168 (87.0) | 320 (86.3) | 0.952 |
| Potent P2Y12 inhibitor (%) | 30 (13.1) | 36 (13.9) | 27 (11.7) | 25 (13.0) | 51 (13.7) | 0.954 |
| Statin (%) | 207 (95.0) | 228 (97.0) | 205 (96.2) | 169 (93.9) | 343 (98.3) | 0.077 |
| Beta blocker (%) | 183 (92.9) | 225 (96.2) | 200 (94.3) | 169 (96.0) | 307 (94.2) | 0.541 |
| RAS blocker (%) | 149 (65.1) | 176 (68.0) | 160 (69.3) | 133 (68.9) | 271 (73.0) | 0.33 |
| Anticoagulation (%) | 6 (2.6) | 8 (3.1) | 8 (3.5) | 6 (3.1) | 10 (2.7) | 0.981 |
| Glucose-lowering treatment (%) | 121 (52.8) | 166 (64.1) | 141 (61.0) | 134 (69.4) | 265 (71.4) | <0.001 |
| Insulin (%) | 8 (3.5) | 18 (7.0) | 15 (6.5) | 18 (9.4) | 50 (13.6) | <0.001 |
| Metformin (%) | 75 (32.8) | 113 (43.6) | 94 (40.7) | 101 (52.9) | 177 (48.1) | <0.001 |
| Sulfonylurea (%) | 49 (21.4) | 73 (28.3) | 68 (29.4) | 71 (37.2) | 148 (40.1) | <0.001 |
| Thiazolidinedione (%) | 0 (0) | 0 (0) | 0 (0) | 1 (0.5) | 2 (0.5) | 0.496 |
| DPP4 inhibitor (%) | 10 (4.4) | 16 (6.3) | 16 (6.9) | 9 (4.7) | 27 (7.3) | 0.536 |
| α-glucosidase (%) | 7 (3.1) | 13 (5.1) | 13 (5.6) | 16 (8.3) | 30 (8.1) | 0.076 |
| **Angiographic data** |  |  |  |  |  |  |
| Culprit lesion (%) |  |  |  |  |  | 0.618 |
| LM/LAD | 119(51.9) | 120 (46.3) | 110 (47.6) | 96 (49.7) | 175 (47.2) |  |
| RCA/LCX | 110 (48.1) | 139 (53.7) | 121 (52.4) | 94 (50.2) | 196 (52.8) |  |
| LM disease (%) | 20 (8.7) | 8 (3.1) | 11 (4.8) | 12 (6.2) | 19 (5.1) | 0.088 |
| Multivessel disease (%) | 136 (59.4) | 157 (60.6) | 146 (63.2) | 113 (58.5) | 225 (60.6) | 0.889 |
| Complete revascularization (%) | 169 (73.8) | 172 (66.4) | 155 (67.1) | 139 (72.0) | 266 (71.7) | 0.291 |
| Total stent number | 1.7 ± 0.9 | 1.6 ± 0.9 | 1.7 ± 0.8 | 1.6 ± 1.0 | 1.7 ± 1.0 | 0.451 |
| Mean stent diameter | 3.2 ± 0.4 | 3.2 ± 0.4 | 3.1 ± 0.4 | 3.1 ± 0.4 | 3.1 ± 0.4 | 0.079 |
| Total stent length | 34.7 ± 23.0 | 34.6 ± 20.7 | 33.8 ± 18.5 | 35.9 ± 23.1 | 37.0 ± 23.5 | 0.429 |

Values are reported as n (%), mean ± SD, or median [interquartile range]. HbA1c, glycated hemoglobin A; BMI, body mass index; STEMI, ST-elevation myocardial infarction; NSTEMI, Non-ST-elevation myocardial infarction; BP, blood pressure; MI, myocardial infarction; PCI, percutaneous coronary intervention; CABG, coronary artery bypass surgery; LV EF, left ventricular ejection fraction; eGFR, estimated glomerular filtration rate; HDL, high-density lipoprotein; LDL, low-density lipoprotein; GRACE, Global Registry of Acute Coronary Events; GNRI, Geriatric Nutritional Risk Index; RAS, renin angiotensin system blocker; PPAR, peroxisome proliferator-activated receptor; DPP4, Dipeptidyl Peptidase-4; LM, left main; LAD, left anterior descending artery; RCA, right coronary artery; LCX, left circumflex artery

**Table S5. Hazard Ratios and 95% CIs of mortality according to the glycated hemoglobin level in patients after excluding individuals who died in the first 1 year after the last HbA1c measurement**

| HbA1c group | **Unadjusted HR** | | | **Model 1** | | | **Model 2** | | | **Model 3** | | |  |
| --- | --- | --- | --- | --- | --- | --- | --- | --- | --- | --- | --- | --- | --- |
|  | HR | 95% CI | p value | HR | 95% CI | p value | HR | 95% CI | p value | HR | 95% CI | p value | |
| **6.5% <HbA1c ≤7.0%** | Ref. |  |  | Ref. |  |  | Ref. |  |  | Ref. |  |  | |
| **HbA1c ≤6.5%** | 3.69 | 1.2-11.31 | 0.022 | 3.71 | 1.21-11.4 | 0.022 | 3.10 | 1-9.6 | 0.050 | 2.91 | 0.94-9.03 | 0.064 | |
| **7.0% <HbA1c ≤7.5%** | 3.38 | 1.1-10.38 | 0.033 | 3.62 | 1.18-11.12 | 0.025 | 3.54 | 1.15-10.88 | 0.027 | 2.86 | 0.92-8.91 | 0.070 | |
| **7.5% <HbA1c ≤8.0%** | 2.46 | 0.72-8.41 | 0.151 | 2.83 | 0.82-9.75 | 0.099 | 3.08 | 0.89-10.68 | 0.076 | 3.13 | 0.9-10.84 | 0.072 | |
| **HbA1c >8.0%** | 3.36 | 1.15-9.82 | 0.027 | 4.21 | 1.42-12.52 | 0.010 | 4.36 | 1.46-13.03 | 0.008 | 4.16 | 1.39-12.46 | 0.011 | |

Model 1: Hazard ratios by categorized HbA1c adjusted by age, sex, and body mass index. Model 2: Hazard ratios by categorized HbA1c adjusted by model 1 plus systolic blood pressure, hypertension, current smoke, chronic kidney disease, and previous stroke. Model 3: Hazard ratios by categorized HbA1c adjusted by model 2 plus the type of myocardial infarction, left ventricular ejection fraction, low density lipoprotein cholesterol, GRACE score, and complete revascularization. HbA1c, glycated hemoglobin A; HR, hazard ratio; CI, confidence interval; Ref., reference

**Table S6. Hazard Ratios and 95% CIs of mortality according to the glycated hemoglobin level in patients after exclusion according to age**

|  | Age < 65 years | |  | Age >= 65 years | |  |
| --- | --- | --- | --- | --- | --- | --- |
| HbA1c group | HR | 95% CI | p value | HR | 95% CI | p value |
| **6.5% < HbA1c <= 7.0%** | ref |  |  |  |  |  |
| **HbA1c <=6.5%** | 1.48 | 0.23-9.76 | 0.682 | 4.83 | 1.03-22.64 | 0.045 |
| **7.0% < HbA1c <= 7.5%** | 2.82 | 0.54-14.88 | 0.221 | 3.08 | 0.62-15.33 | 0.169 |
| **7.5% < HbA1c <= 8.0%** | 0.92 | 0.11-7.33 | 0.935 | 5.83 | 1.1-31.07 | 0.039 |
| **HbA1c > 8.0%** | 2.27 | 0.44-11.7 | 0.327 | 7.31 | 1.56-34.18 | 0.011 |

HbA1c, glycated hemoglobin A; HR, hazard ratio; CI, confidence interval; Ref., reference

**Table S7.** **Baseline characteristics of diabetic patients who were excluded from this study and included in this study**

|  | **Excluded patients (N=2709)** | **Included patients (N=1384)** | **p** |
| --- | --- | --- | --- |
| Mean HbA1c level | 7.5 ± 1.6 | 7.6 ± 1.2 | 0.015 |
| Number of HbA1c measurement | 1.1 ± 0.7 | 5.4 ± 2.9 | <0.001 |
| Mean follow-up period, years | 1389.3 ± 1123.4 | 2345.1 ± 854.3 | <0.001 |
| Age (years) | 67.2 ± 11.5 | 60.3 ± 11.2 | <0.001 |
| Male (%) | 1714 (63.3) | 1012 (73.1) | <0.001 |
| BMI (kg/m^2^) | 24.0 ± 3.4 | 24.9 ± 3.2 | <0.001 |
| Diagnosis (%) |  |  | 0.237 |
| STEMI | 1353 (49.9) | 719 (52.0) |  |
| NSTEMI | 1356 (50.1) | 665 (48.0) |  |
| Cardiac arrest on arrival (%) | 39 (1.4) | 6 (0.4) | 0.006 |
| Systolic BP (mmHg) | 127.9 ± 27.4 | 131.8 ± 27.3 | <0.001 |
| Diastolic BP (mmHg) | 77.6 ± 17.0 | 79.7 ± 16.6 | <0.001 |
| Heart rate (bpm) | 82.0 ± 20.2 | 80.6 ± 19.1 | 0.039 |
| Killip class ≥3 (%) | 565 (20.9) | 140 (10.1) | <0.001 |
| Hypertension (%) | 1781 (65.7) | 828 (59.8) | <0.001 |
| Dyslipidemia on drug treatment (%) | 477 (17.6) | 244 (17.6) | 1 |
| Current smoker (%) | 836 (30.9) | 562 (40.6) | <0.001 |
| History of MI (%) | 166 (6.1) | 43 (3.1) | <0.001 |
| History of PCI (%) | 285 (10.5) | 81 (5.9) | <0.001 |
| History of stroke (%) | 271 (10.0) | 92 (6.6) | <0.001 |
| History of Cancer (%) | 98 (3.6) | 38 (2.7) | 0.168 |
| LV EF, % | 51.1 ± 12.3 | 52.9 ± 11.0 | <0.001 |
| Total cholesterol (mg/dL) | 171.2 ± 46.4 | 176.7 ± 44.0 | <0.001 |
| Triglyceride (mg/dL) | 126.9 ± 90.3 | 149.5 ± 119.2 | <0.001 |
| HDL cholesterol (mg/dL) | 39.8 ± 11.7 | 39.7 ± 10.1 | 0.698 |
| LDL cholesterol (mg/dL) | 107.1 ± 40.0 | 110.0 ± 37.5 | 0.031 |
| eGFR (ml/min/1.73m2) | 66.4 ± 29.5 | 76.0 ± 26.3 | <0.001 |
| Dialysis (%) | 148 (5.5) | 25 (1.8) | <0.001 |
| eGFR ≤ 30 (%) | 378 (14.0) | 93 (6.7) | <0.001 |
| 30 < eGFR≤ 60 (%) | 698 (25.8) | 248 (17.9) | <0.001 |
| GRACE score | 153.0 [129.0, 182.0] | 133.0 [111.0, 154.0] | <0.001 |
| GNRI | 95.1 ± 17.3 | 97.7 ± 10.5 | <0.001 |
| **Discharge medication** |  |  |  |
| Aspirin (%) | 2431 (89.7) | 1366 (98.7) | <0.001 |
| Clopidogrel (%) | 2147 (79.3) | 1211 (87.5) | <0.001 |
| Potent P2Y12 inhibitor (%) | 317 (11.7) | 172 (12.4) | 0.531 |
| Statin (%) | 2191 (80.9) | 1230 (88.9) | <0.001 |
| Beta blocker (%) | 2040 (75.3) | 1156 (83.5) | <0.001 |
| RAS blocker (%) | 1946 (71.8) | 1088 (78.6) | <0.001 |
| Anticoagulation (%) | 85 (3.1) | 41 (3.0) | 0.833 |
| Glucose-lowering treatment (%) | 1378 (50.9) | 881 (63.7) | <0.001 |
| Insulin (%) | 223 (8.4) | 125 (9.1) | 0.486 |
| Metformin (%) | 822 (30.9) | 586 (42.6) | <0.001 |
| Sulfonylurea (%) | 685 (25.8) | 428 (31.1) | <0.001 |
| Thiazolidinedione (%) | 6 (0.2) | 3 (0.2) | 1 |
| DPP4 inhibitor (%) | 151 (5.7) | 84 (6.1) | 0.628 |
| α-glucosidase (%) | 159 (6.0) | 86 (6.2) | 0.775 |
| **Angiographic data** |  |  |  |
| Culprit lesion (%) |  |  | 0.037 |
| LM/LAD | 1383 (51.0) | 676 (48.8) |  |
| RCA/LCX | 1320 (48.7) | 708 (51.1) |  |
| LM disease (%) | 209 (7.7) | 82 (5.9) | 0.041 |
| Multivessel disease (%) | 1629 (60.1) | 854 (61.7) | 0.249 |
| Complete revascularization (%) | 1842 (67.9) | 963 (69.6) | 0.016 |
| Total stent number | 1.6 ± 1.0 | 1.7 ± 0.9 | 0.124 |
| Mean stent diameter | 3.1 ± 0.4 | 3.1 ± 0.4 | 0.027 |
| Total stent length | 36.0 ± 22.4 | 35.4 ± 21.7 | 0.387 |

Values are reported as n (%), mean ± SD, or median [interquartile range]. HbA1c, glycated hemoglobin A; BMI, body mass index; STEMI, ST-elevation myocardial infarction; NSTEMI, Non-ST-elevation myocardial infarction; BP, blood pressure; MI, myocardial infarction; PCI, percutaneous coronary intervention; CABG, coronary artery bypass surgery; LV EF, left ventricular ejection fraction; eGFR, estimated glomerular filtration rate; HDL, high-density lipoprotein; LDL, low-density lipoprotein; GRACE, Global Registry of Acute Coronary Events; GNRI, Geriatric Nutritional Risk Index; RAS, renin angiotensin system blocker; PPAR, peroxisome proliferator-activated receptor; DPP4, Dipeptidyl Peptidase-4; LM, left main; LAD, left anterior descending artery; RCA, right coronary artery; LCX, left circumflex artery

**Figure S1. Cubic spline curve showing the hazard of all-cause mortality according to mean HbA1c.** Solid lines denote the calculated log relative hazard ratios, and the shaded area denotes the 95% confidence intervals. Hazard ratios for all-cause mortality were adjusted for age, sex, body mass index, systolic blood pressure, hypertension, current smoke, chronic kidney disease, previous stroke, the type of myocardial infarction, left ventricular ejection fraction, low-density lipoprotein cholesterol, GRACE score, and complete revascularization.

**
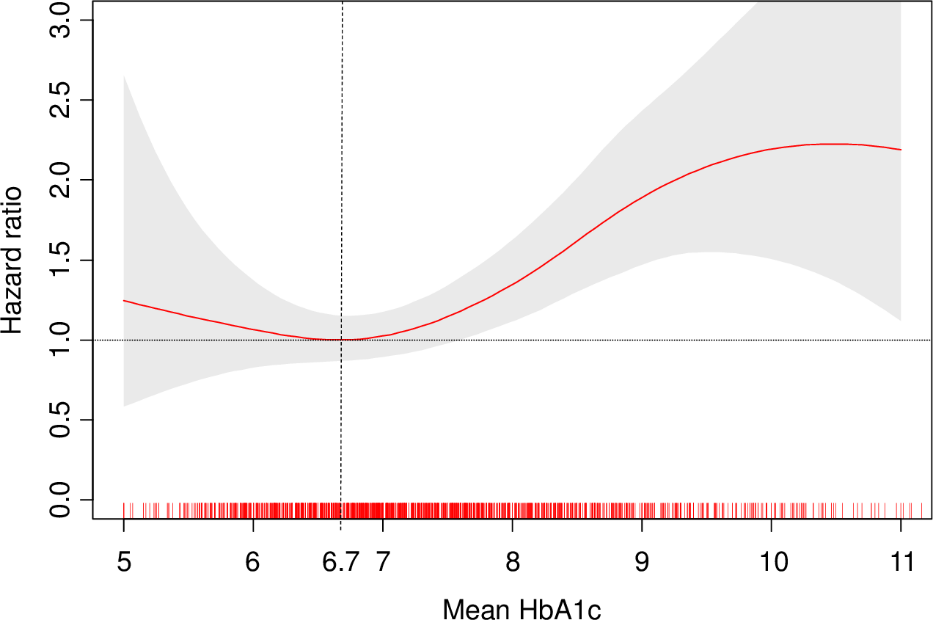
**
